# Risks and Crises for Healthcare Providers: The Impact of Cloud Computing

**DOI:** 10.1155/2014/524659

**Published:** 2014-02-20

**Authors:** Ronald Glasberg, Michael Hartmann, Michael Draheim, Gerrit Tamm, Franz Hessel

**Affiliations:** SRH Hochschule Berlin, 10587 Berlin, Germany

## Abstract

We analyze risks and crises for healthcare providers and discuss the impact of cloud computing in such scenarios. The analysis is conducted in a holistic way, taking into account organizational and human aspects, clinical, IT-related, and utilities-related risks as well as incorporating the view of the overall risk management.

## 1. Introduction

In the industrialized countries hospitals are the backbone of the healthcare system. In Germany 18.620.422 hospital treatments were conducted in 2.017 hospitals in 2012 [1]. Like in most countries, nearly half of the hospital beds are in public ownership with a growing number of privately owned hospitals [2]. The aim of the hospitals is to heal diseases, prevent their deterioration, or alleviate disease symptoms, with specialized staff and equipment. For that reason, hospitals are a relatively hazardous working environment for patients as well as staff. The hospital staff has to deal with adverse events and numerous potential, for example, wound infections, medication errors, and wrong-site surgery [3, 4]. This permanent risk of unsafe situations makes the hospital sector an important setting for an assessment for safety and risk management. The majority of the publications and studies on risk management in hospitals addressed clinical safety and risk management in specific indications, medical subspecialties, or treatment settings such as intensive care or operation theatre [5–7]. Despite this substantial body of research in the area of patient safety in specific situations there are only a small number of systematic reviews or comprehensive, interdisciplinary approaches. Based on a systematic literature review Hoff and colleagues postulated that primarily the interventions and not the organizational structure and features are linked directly to patient safety [8]. In a more recent work, Dückers and colleagues draw the somehow frustrating conclusion that the scientific evidence for safety interventions in hospitals still is limited and that the methodological quality of the studies is generally weak [9]. Although a recent hospital survey indicates increased attention to the management of risks in hospitals, we are far from having defined a general approach for various sources of risks, their analysis, evaluation, and treatment [10].

Not only the risks directly related to patient treatment, but also the continuous governmental healthcare reform acts and the increasing financial pressure on healthcare spending are big challenges for a sustainable hospital management. On the other side, information technology innovations are often considered as a major factor for the improvement of quality, efficiency, and efficacy in healthcare [11]. As one approach electronic health records (EMR) promise to improve efficiency and effectiveness of healthcare providing processes [12]. The use of electronic data in hospitals is ubiquitous and inevitable and the use of health IT is still increasing but according to the most recent data still only less than one-third of the hospitals in the US use a kind of electronic medical records [13]. Due to the slow speed of implementation of information technology the expected massive cost savings by EMR did not yet come true [14]. With regard to the quality of patient care there is only marginal improvement, too [15]. In particular, medical doctors seem to be relatively reluctant to leave the traditional way of unstructured paper-pencil documentation [16] and to adopt IT technologies in daily patient care. Other approaches go as far as incorporating virtual or mixed reality [17] as well as intelligent systems [18] in healthcare scenarios. Cloud computing (CC) is increasingly being viewed as a key innovation in this regard and is generally considered one of the most important developments in IT [19]. But in addition to the opportunities that information technology pervasion to a hospital, these new technologies also pose risks to the organisations. Security and privacy are the relevant threats for hospitals in such a cloud environment, because health data are the most private and sensitive data about the patients [20].

In our project, we extended the scope of the potential use of health IT and cloud computing in hospitals, from the “classical” objectives of cost savings, quality management, and clinical risk management to hospital crises. Objectives of the project are to identify the specific crisis scenarios perceived as most relevant by hospital care providers, to evaluate the preparedness of hospitals to prevent, respectively, handle the crisis scenarios, and to describe and develop IT and cloud computing solutions to support crisis management in hospitals. The specific focus of this paper lies on identification and handling of IT crises.

In general, a crisis is described as “an abnormal situation, or even perception, which is beyond the scope of everyday business and which threatens the operation, safety, and reputation of an organisation” [21]. Transferred to hospital management, a crisis is one or numerous critical situations which could not be handled by routine measures of quality management. A hospital crisis is regarded as an event or a series of events, which may occur either suddenly or which may take some time to evolve. It results in a major, urgent problem with potentially severe consequences for the hospital and it must be addressed immediately.

**Figure 1:**
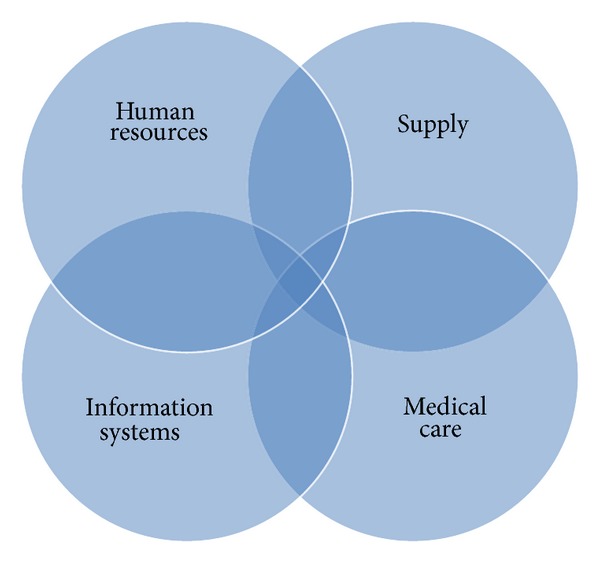
Considered areas in a hospital.

Hospital crises can roughly be categorized into natural disasters (i.e., earthquakes, floods, or fires), significant operational problems (i.e., personnel emergencies, accidents, and theft of proprietary data) or extraordinary problems (i.e., hostage situations) [22]. To identify all relevant crises to a hospital, it is also necessary to address internal problems. As visualized in Figure 1, we classified hospital crises into four areas according to the professional disciplines affected by the crisis: medical care, information systems (IS), human resources (HR), and supply. In a contribution with the use of CC in hospitals we present our results from the area of Information Systems and Supply.

The rest of this work is structured as follows. In Section 2 risks associated with IT and utilities are discussed.  Section 3 gives an overview of the overall approach and in Section 4 the results of the evaluation are presented. Finally in Section 5 we discuss our results and future research activities.

## 2. IT-Related and Utility-Related Risks

When running IT systems which process health data, both the original organization (e.g., a clinic) and the CC provider should implement a number of appropriate technical and organizational measures of precaution. For data originating from German healthcare applications these measures are specified in a catalog of eight control requirements (§ 9 in conjunction with the annex to § 9 BDSG—the German Data Protection Law). There is a similar requirement for socially related data in § 78a of the Social Codex (Sozialgesetzbuch, SGB), in conjunction with the annex to § 78a SGB.

Associated risks in this context arise from the fact that the law stipulates only general requirements. The precise definition and implementation of specific measures are the obligation of both the healthcare provider and the CC provider. For example, both should apply general measures for protecting personal data (e.g., limited access) when dealing with health-related data and also implement measures to protect data transmission (e.g., encryption). Furthermore, systems that are operated for more than one client (e.g., processing appointment data or analysis data for multiple clinics) should ensure strict separation between data of each client organization. There exist specific recommendations about the compliant operation of a hospital management system (HIS) [23]. Similar requirements apply for CC and outsourcing scenarios, as providers are expected to implement and assure security requirements of the client organization.

Specific risks and crises can occur when the organization is not capable to follow all applicable regulations in the area of medical confidentiality, social data, and state-specific rules (rules that are different in every specific German federal state).

Medical confidentiality describes the relation of trust between a doctor and a patient. In Germany it is regulated in the professional code of conduct for doctors (Muster-Berufsordnung für die deutschen Ärzte und Ärztinnen (MBO-Ä)) with medical confidentiality specified in § 9 Section.1 MBO-Ä. A breach of confidentiality is considered a criminal offense and a reveal of patient data can result already from archiving patient data with a service provider without the previous written consent of the patient. This previous written consent should include the specific data and the legal information about the service provider and is therefore often unfeasible.

Social data as a term includes all personally related data that concerns social aspect of a person. The increased confidentiality requirements for social data are defined in § 35 Sect. 1 SGB I. A specific example of regulations in this area is the recently introduced “electronic health card” (elektronische Gesundheitskarte, eGK), a reduced EMR backup on the identity card of statutory sickness fund enrollees. Requirements concerning data protection in the context of the eGK are specified in Volume V of SGB, with particular regulations concerning encryption and access control lists (ACLs) in § 291a SGB V.

Further IT-related risks and crises can also occur when a clinic neglects obligations mandated by state-specific rules (e.g., specific and different rules in Bavaria, Hamburg, or Berlin) with respect to data protection and information processing. A variety of state-specific hospital laws exist that often stipulate different requirements with respect to patient data processing. For example, according to the state hospital law (Landeskrankenhausgesetz, LKG) of Berlin, hospitals in Berlin are either allowed to process patient data in-house or outsource this process to another hospital. Other providers can process patient data under the mandate of the hospital only if it has been sufficiently anonymized in order to eliminate person-related aspects from it (§ 24 Sect. 7 (2) LKG Berlin).

Data processing in the context of CC typically constitutes the so called data processing under mandate (German: Datenverarbeitung im Auftrag) as stipulated by § 11 BDSG. This results in another wide range of risk and crisis-scenarios stemming from the specific requirements regarding the contractual relationship between client and service provider. The contract should specify the type and scope of the intended use of data, the control rights of the client, and the specific technological and organizational measures that the provider will be implementing in accordance with § 9 BDSG. Furthermore, prior to the start of the actual data processing under mandate, the client has to carefully select the service provider and to convince himself that the technological and organizational measures are appropriate (§ 11 Section 2 (4) BDSG). This control obligation continues during the actual data processing with a requirement of regularly controls. Noncompliance with it can result in regulatory fines. Major CC providers in Germany conduct yearly audits by independent audit organizations and make the audit reports available to their clients (http://www.pironet-ndh.com/site/pndh-website-site/node/269414/Lde/).

Data processing under mandate with respect to social data is regulated similarly but by § 80 SGB X. There are several important differences to § 11 BDSG that can lead to additional risk and crisis scenarios—client organizations are allowed to use in general only providers from the public administration. The inclusion of a private CC provider can only be considered if otherwise there will be substantial problems for the normal operation of the client organization or if there are substantial cost benefits in comparison to a provider from the public administration. As there are currently no reliable assessments whether private CC providers can plausibly meet these conditions, we regard their inclusion in scenarios covered by the SGB as legally unclear and thus having the potential to further amplify the impact of major IT-related risks and crises scenarios.

The presented inherent risks of cloud-based data processing for healthcare providers show that these organizations should have an elaborated demand and requirements concept with respect to data privacy. The concept should consider aspects such as the selection and evaluation process of possible CC providers, specific detailed requirements about service level agreements (SLAs), and specifically required organizational and technical measures that the CC provider should conform to. This dramatically increases transaction costs in the CC market, which is already marked by high levels of information asymmetry [24]. Some existing automated approaches for matching demand and supply, even at the level of SLAs [25], are only of limited benefit, as they cannot account properly for complex organizational measures. Specific technical measures, on the other side, can be clearly stated in automated supply statements (e.g., the so called service level objectives as introduced in [26]) and can therefore be easily matched to automated requirements. Recommendations for specific measures can be derived from relevant standards, such as the Baseline IT-Security (IT-Grundschutz) standard by the Federal Office for Information Security (Bundesamt für Informationssicherheit, BSI).

Utilities-related risks associated with operating a healthcare provider have been rarely studied, with power-supply-related incidents being considered only during intra-hospital transfer of critically ill patients [27]. Other important utilities, for example, the supply with gases have only been considered in the context of standardization efforts for the specific case for Britain in 1979 [23], while water supply is typically assessed only as a potential source of infections (e.g., Legionellaceae) [26, 28, 29].

In our approach, we introduce the two perspectives, the IT-related and the utilities-related, into the overall model in order to better estimate the impact of crises that can arise from these fields and to better recommend appropriate countermeasures, both proactively and retroactively.

In this work we present an approach that aims to consider diverse aspects in the context of risk and crisis management for healthcare providers in the context of CC, ranging from human resources and clinical management to IT-related and utilities-related aspects. Our analysis is focused on Germany, as it is a jurisdiction with one of the most elaborated and restrictive regulations with respect to liability, data protection, and duty of care particularly in the area of healthcare [30].

## 3. Overview of the Approach

The objective of our project, “Risk Management in hospitals”, is to analyze the behavior of the various key players in the fields of Medical Care and Medicine, Supply, HR, and IT-Systems with regard to the influence of dynamic risks in the context of various simulated scenarios. In the first step of the research project, a network of experts and executives from politics, business, and media related to the hospital field should be established, accompanied by the creation of a literature database. In a second step, information will be collected in expert workshops and, in combination with the results of the literature search and the expert interviews, will be used for the conception, planning, and implementation of a prototype Decision-Making Tool. With such a tool (based upon artificial neural networks), safety-relevant deficits in the hospital as well as the development of ideal-solutions will be illustrated. This project will thus provide decision-making support for directing and managing bodies of hospitals. Our empirical assessment follows a qualitative approach. For the most important hospital crises, identified by literature search and interdisciplinary expert groups, we evaluate the preparedness of German hospitals and develop adequate management scenarios including IT solutions such as cloud computing. These solutions are used for on-site approaches to avoid incidents, to exchange data, as an information source, for example, for guidance documents as well as active training tools.

The core tools of our project were four expert workshops, one for each cluster of crises in IT, HR, medical care, and utilities. The participants consisted of experts and leaders of the respective fields. For the expert workshops a standardized agenda was set with the purpose of identifying the most important crises of each area. The workshops were structured in five phases, that is, the brainstorming phase, the discussion and precision phase, the evaluation phase, the dyadic phase, and the presentation phase. In the brainstorming phase the experts and managers were asked to write down all the crises they could think of. Following this, the identified crises were written on cards and clustered on pin boards by the workshop leaders. In the second phase the identified crises were presented and discussed in the whole group. The workshop leaders for the respective areas shortly presented the crisis given by the group and discussed possible ambiguities. After all, participants had the same level of knowledge about the identified crises. All of them were asked to select the most important crises from the first brainstorming in the third phase. For this purpose, each of the participants had the opportunity to award 10 points, with a maximum of 5 points for one crisis. By this vote the total number of collected crises was reduced.

In the fourth phase, the workshop participants were divided into teams of two experts. Each team of two should choose to edit two crises. The processing was done by dyadic interaction, where the two experts first worked out key features, consequences, and costs of a crisis and fixed the results on a poster. The teams had 45-minute time for the development of a single crisis. In the final workshop phase, the results of the teamwork phase were presented. For this purpose, each team introduced their findings to the group and then the results of the dyadic phase were discussed together. In the last step, the participants received the possibility to rate the danger and the probability of occurrence of the crises by setting points to a prepared evaluation scale on the posters. The final assessments served to produce a better ranking of crises.

In order to acquire participants for the workshop, a representative sample of German hospitals equally distributed with regard to ownership, number of beds, and level of care of the average population was determined. After preliminary phone calls with the managers or their assistants of hospitals (*n* = 195) personal invitations were sent out. Overall, a number of (*n* = 16) experts attended each of the workshops: WS 1 consisted of Medical Care and HR and WS 2 was about Information Systems and Supply. In WS 1 physicians and experts from hospital management and quality management participated. WS 2 was attended especially by heads of the IT departments as well as technical directors of hospitals.

## 4. Results

A number of specific crises in hospitals were characterizing the debates in the workshops. In particular in the area of medical malpractice, the “Use of medical devices or implants with defects or insufficient approval” and the “Occurrence of hygiene crises due to organizational deficits” were highlighted by the participants among others. All hospitals are threatened periodically by these problems which pose a significant risk to the economic survival. The fact that the participants (rather from the medical field) consider the crisis “Failure of the edp system” as one of the top-rated five crises from the field of Medical Care underlines the increasing importance of information systems in health care.

In the second expert workshop, the major crises were collected from the field of information systems and categorized according to their impact on hospitals. The results are shown in Table 1. In particular, the failure of the information technology infrastructure was identified as crisis. Furthermore, it may be discerned that the threat of cybercrime such as trojans, viruses, and also social hacking poses a relevant threat to the hospitals. Other major crisis scenarios resulting from menace arise from the treatment of patients. Also in the workshop with participants primarily from the information technology area some crises that affect the IT-support of patient treatment were identified.

Another important aspect within hospital crisis management is the dependence on a variety of external resources. As shown in Chapter 1 hospitals are not only crisis-prone, they also depend on a variety of critical infrastructures. This results in a crises-evaluation in the field of supply which is shown in Table 2.

The energy supply in hospitals is an element that requires a precise control, because a current reduction for some minutes or a blackout could have a significant impact due to inoperative medical equipment, hampered communications and transportation, stopped heating, and water supply. All scenarios could generate a collapse in the services. Hospitals wouldn't be able to work if they do not have a process to counter the interruption; for this reason, it is important to have a plan to mitigate and counter any emergency and also to reduce any potential risk. The “Loss of power for more than 48 hours” was highlighted by the participants as particularly important. Thus, existing fuel reserves have only to ensure the operation up to 24 hours [31]. Other key points from this workshop field were an outbreak of “fire” and the “Spills of dangerous substances”. When these events occur they have a significant impact on hospitals.

## 5. Discussion and Outlook

The consideration of hospital crises in the context of cloud computing has the potential to bring new insights to decision makers in healthcare and also to enhance the body of knowledge both in the areas of healthcare management and IT management. Furthermore, by pursuing a holistic approach our work offers a framework where the implications of crises can be considered for the whole organization. The approach defines hospital crisis as an event or a series of events, which may occur either suddenly or which may take some time to evolve. It results in a major, urgent problem with potentially severe consequences for the hospital and it must be addressed immediately. The selected evaluation methodology—expert workshops—is an established approach, particularly in the area of health-related research [32]. The identified crises during the evaluation confirm expectations that problems with cloud-based systems (and IT systems in general) can lead to substantial limitations of the handling capability of a hospital. In order to further corroborate these findings the research team has launched a broad survey of hospital managers in German-speaking countries. Although the survey is focused on Germany as the largest healthcare market in Europe the results are considered to be exemplary and generic to other European countries as they reflect crisis scenarios described in the literature [9]. To our knowledge there is no preceding project presenting a systematic evaluation of crisis management in hospitals differentiating the described areas. As a next step the survey is going to be active until the first quarter of 2014 and authors expect to submit results from it for publication in the second half of 2014. Based on the findings of the expert workshops and the expert survey authors plan to develop a decision-support-tool that will extend the capabilities of standard risk assessment methods with the presented holistic and domain-specific approach and thus provide hospital managers with a tailored instrument for crisis mitigation and aversion.

## Figures and Tables

**Table 1:** Results from WS 2—Information systems.

Rank	Field	Risk/crisis	Description
1	IS	Failure of the entire IT infrastructure, or of individual parts	The failure of the IT structures leads to the disturbance of the normal hospital workflow. The necessary flow of information is interrupted. Doctors and nurses cannot access important treatment information (such as laboratory test results). The administration cannot access rosters and accounting data.

2	IS	Trojan, virus, hacking	A criminal and defective attack on the information systems of a hospital has taken place. The data of the patient/hospital were copied, destroyed or damaged. The attacks are not or at a later timepoint noticed. The privacy of the patient is injured. Legal implications for the hospital might occur, if it is not proven that all necessary protective measures have been made.

3	IS	Application systems are not available	Application systems, which are necessary for adequate treatment of patients, such as the hospital information system (HIS) cannot be accessed. The technical staff of the hospital is unable to solve problems within a short time. The information (such as diagnostic images) required by the medical staff are not available. There are some limitations in the treatment as well as adequate performance documentation.

4	IS	Data theft/Social Hacking	Social Hacking is the acquisition of information through manipulation and deception of a person. Employees and partners have access to highly sensitive data. This approach is performed directly or through third parties. Because of carelessness or criminal activity, these data become public. For the hospital it means creating a large image damage and loss of reputation.

5	IS	Poor ergonomics lead to incorrect entries/interpretations	Poor software ergonomics lead to incorrect entries or misinterpretation of clinical data of patients. It can increase the appearance of incorrect entries. Due to outdated systems, the probability of incorrect entries or misidentification may still increase. There will be mistreatment of patients by the poor software ergonomics.

**Table 2:** Results from WS 2: supply.

Rank	Field	Risk/crisis	Description
1	Supply	Loss of power for more than 48 hours	There is a power failure lasting longer than 48 hours. The propellant and thus the emergency power supply cannot be maintained over the entire duration of the power failure. It comes to a gradual failure of all supply elements (e.g., hot water, heating, and cooling) and communication (within and outside of the hospital). The treatment can be carried out only in a severely restricted way or not at all.

2	Supply	Heating/air falls out: evacuation necessary	Due to a failure of the heating or cooling system, an evacuation of the hospital is necessary. In consequence of a very short time frame and the threat of patient risk, an immediate action is needed. It comes to a mismatch between existing and required human resources. Scheduled treatment cannot take place and the hospital is no longer accepting patients.

3	Supply	Fire (smoke on ward)	A fire spreads out at a unit with the consequence of smoke and fire damage. Patients and staff are at risk. The unit has to be evacuated.

4	Supply	Failure of the water supply	In health care facilities such as hospitals, the availability of drinking water is essential to survive. A supply of water in the hospital cannot be ensured. The use of sanitary units and the execution of cleaning operations are no longer possible. While the remedy no medical processes can take place. Depending on the duration and extent of the failure, the hospital has to be evacuated.

5	Supply	Spills of dangerous substances (e.g., chlorine gas)	In many functional units of the hospital hazardous substances are used daily. These include for example disinfectants, surgical gases, drugs, and chlorine gases. There will be a release of these substances in larger quantities. The station is contaminated and needs to be evacuated. Patients and staff are directly at risk.
